# Technical and Clinical Validation of a Portable Optical Fibre Balance Mat for Quantifying Postural Sway in Older Adults

**DOI:** 10.3390/s26134021

**Published:** 2026-06-24

**Authors:** Abishek Shrestha, Damith Herath, Angie Fearon, Maryam Ghahramani

**Affiliations:** 1 Faculty of Science and Technology, University of Canberra (UC), Bruce, ACT 2617, Australia; damith.herath@canberra.edu.au (D.H.); maryam.ghahramani@canberra.edu.au (M.G.); 2 Faculty of Health, University of Canberra, Bruce, ACT 2617, Australia; angie.fearon@canberra.edu.au

**Keywords:** technical validity, clinical validity, discriminative validity, force plate, postural sway

## Abstract

**Background:** Early identification of balance impairments is critical for detecting the fall risk in older adults. Force plates are the standard for measuring postural sway, but are restricted in practice because they are cumbersome and expensive. The Balance Mat is a portable device that requires comprehensive validation against force plates and clinical benchmarks in older adult populations. **Objective:** The objective of this study was to evaluate the technical validity and clinical discriminative ability of the Balance Mat against a laboratory-grade force plate, clinical tests, and the fall history in an older adult cohort. **Methods:** Fifty-six community-dwelling older adults performed static balance assessments across six stance conditions. Postural sway data were recorded simultaneously using the Balance Mat and a force plate. The technical validity was assessed using Spearman’s rank correlation and intraclass correlation coefficients. Linear regression models were applied to calibrate the Balance Mat outputs against the force plate. The diagnostic accuracy for classifying the fall risk against the timed up and go test, the Falls Efficacy Scale-International, and the retrospective fall history was evaluated using an area-under-the-curve analysis. **Results:** The Balance Mat demonstrated strong associations with force plate measurements, particularly for the sway path/velocity, variance and Area95 (r>0.80). Following calibration, the absolute agreement for the sway path/velocity reached excellent levels (ICC=0.93) and good levels for Area95 and RMS (ICC>0.75), whereas the mean sway demonstrated a poor agreement and was excluded. For fall-risk classification, the calibrated Balance Mat achieved a fair accuracy for the retrospective fall history and a high Falls Efficacy Scale-International concern (area under the curve, 0.76–0.78), and a fair accuracy for the timed up and go thresholds (area under the curve, 0.70). **Conclusions:** The calibrated Balance Mat provided valid measurements of postural sway that aligned with the force plate parameters, particularly for the sway path/velocity and Area95. The within-stance agreement for the sway path/velocity ranged from ICC = 0.44 to 0.88, and the pooled value should not be interpreted as the uniform performance across all stance conditions. Given its fair diagnostic accuracy, the device is best utilised as a portable screening tool in combination with standard clinical assessments and the fall history rather than as a standalone diagnostic test.

## 1. Introduction

Falls among older adults represent a major global public health concern, contributing substantially to morbidity, mortality, and healthcare costs [1,2,3]. In Australia, falls were the leading cause of both injury-related hospitalisations and deaths in 2023–2024, accounting for 43% of all injury cases and costing the healthcare system more than $5 billion (AUD) annually [1]. Individuals aged 65 years and older experience the highest rates of fall-related injuries and fatalities, which creates a major physical, psychosocial, and economic burden [1,3,4,5]. The early identification of balance impairments is essential for detecting the fall risk before an incident occurs [1,2,6].

There are different methods of assessing the risk of falls, which include clinical and instrumented tests. Clinical tests, such as the Berg balance scale [7], the timed up and go test [8], and the Tinetti balance test [9], are commonly used because they are simple and familiar to clinicians. However, they rely on observer scoring and show ceiling effects in higher-functioning adults [3,10]. To address the limitations of clinical tests, instrumented tests provide an alternative by measuring postural sway, which has been reported to be indicative of the risk of falls [2,3].

Among instrumented tests, the force plate (FP) is considered the gold standard for postural sway assessments and measuring the centre of pressure (CoP) displacement [3,11,12]. However, it is limited in practice because it is expensive, mostly non-portable, requires dedicated space and power, and usually needs expert personnel to operate and interpret [3,12]. There has been an increase in the use of portable alternatives such as the Nintendo Wii Balance Board and wearable inertial measurement units (IMUs), which have been tested against the FP [3,13]. However, there are limitations on how they have been validated, as they do not report on the association, agreement and discriminative validity against the clinical tools in a single protocol.

In response to the limitations of the current devices, the Balance Mat (BM) has been introduced. The BM offers a distinct technological alternative to other routine balance assessment devices. It uses an optical-fibre layout to detect pressure-induced changes in light transmission [14,15]. Unlike wearable IMUs, the BM relies on foot contact, eliminating the need for body-worn placement and sensor orientation [13,16]. Furthermore, the BM is a Therapeutic Goods Administration (TGA)-approved medical-grade device, whereas the Wii Balance Board was originally developed for consumer use and later adapted for balance assessments [17]. In a study by Ghahramani et al. [14], the BM was compared against the IMU in older adults, and a strong correlation was found. In another study by Raj et al. [18], the reliability and validity were compared against the FP in older adults as a pilot study, and good relative reliability and comparable sway to FP were found. In our previous study, we compared the BM with a laboratory-grade FP in healthy younger adults and found a strong correlation [15]. However, the BM has not been fully validated against the FP in older adults, nor has its discriminative validity been established against clinical tests and a retrospective fall history.

Therefore, this study undertook a comprehensive validation of the BM within an older adult population against the FP, clinical benchmarks and the fall history. We hypothesised that: (1) the BM will demonstrate a strong association with the FP; (2) agreement with the FP will need calibration; and (3) the BM will have a discriminative ability to distinguish high-fall-risk participants as identified by clinical benchmarks. The main contributions of this study are: (1) an assessment of the technical validity of the BM against a gold-standard FP for postural sway measurements in older adults; (2) the determination of the BM’s accuracy in quantifying the sway magnitude following linear regression calibration; and (3) an evaluation of the BM’s clinical discriminative ability to classify participants based on their TUG performance, FES-I scores, and fall history. By establishing these properties, the BM can serve as a valid, low-cost, and portable device for objective balance assessments and fall-risk screening.

## 2. Methods

### 2.1. Instrumentation

The Balance Mat (Balance Mat Pty Ltd., Canberra, Australia) is an Australian-manufactured, TGA-approved portable balance assessment device designed to quantify standing postural sway. The device utilises optical fibres arranged in a grid configuration, with 32–40 crossover points acting as individual pressure-sensing nodes beneath the feet. When pressure is applied to the mat, the resultant compression of the optical fibres alters the intensity of transmitted light. These light intensity variations are detected by an embedded microcontroller and converted into one-dimensional unitless pressure signals representing plantar pressure distribution. The BM records real-time postural sway data at a sampling frequency of 40 Hz. The data are transmitted to a computer via a USB connection, and the device operates without the need for an external power supply. The mat measures 600 mm × 700 mm × 6 mm and weighs approximately 2.5 kg, enabling easy portability for clinical and community-based assessments [14,15].

**Figure 1:**
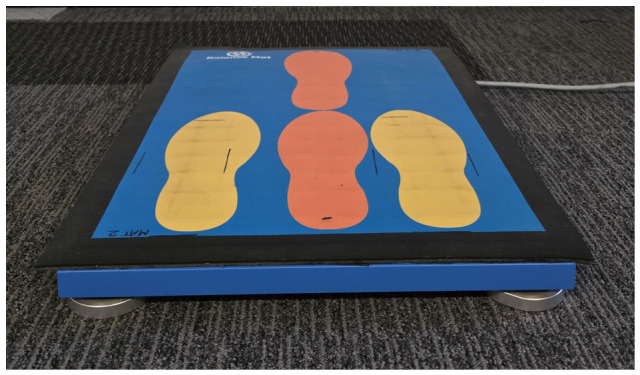
Experimental setup showing the portable Balance Mat positioned on top of the reference laboratory force plate.

A laboratory-grade force plate (Kistler, Model 9260AA6, Kistler Group, Winterthur, Switzerland) was used as the gold-standard reference for postural sway measurements. The FP recorded CoP displacement in both the anterior-posterior (AP) and medial-lateral (ML) directions. To enable simultaneous data acquisition, the BM was positioned directly on top of the FP as shown in Figure 1. Both devices recorded data at a synchronised sampling frequency of 40 Hz to facilitate a direct comparison of sway features.

### 2.2. Participants

Based on our prior study in younger adults [15] that observed a medium Cohen’s q effect size of 0.35, we determined that 46 participants would provide 80% power at α=0.05. After allowing for 15% dropout, the target was 55. To account for potential data loss or exclusion, the recruitment target was set slightly higher, resulting in a slightly higher final sample size. The study cohort consisted of 56 community-dwelling older adults (43 females [76.8%] and 13 males [23.2%]) with a mean age of 75.29 ± SD 6.27 years (range: 65–88 years). The participants had a mean height of 163.99 ± SD 10.23 cm and a mean body mass of 69.94 ± SD 14.77 kg. Based on the self-reported fall history within the previous 12 months, 44 participants (78.6%) were classified as non-fallers, while 12 participants (21.4%) were classified as fallers. Participants were excluded if they had diagnosed neurological disorders, musculoskeletal impairments, lower limb injuries, neuromuscular disorders, or vestibular conditions such as vertigo that could influence their balance performance. All the study procedures were approved by the University of Canberra Human Research Ethics Committee (approval No.: 20249208), and written informed consent was obtained from all the participants before testing.

### 2.3. Clinical Assessments

Prior to the experimental protocol, the participants underwent two clinical assessments to evaluate their functional mobility and fear of falling: the timed up and go (TUG) test and the Falls Efficacy Scale-International (FES-I). These assessments were conducted by a trained assessor following standardised protocols [8,19].

#### 2.3.1. Timed Up and Go (TUG) Test

The TUG test was administered to assess functional mobility and dynamic balance [8]. The participants were instructed to stand up from a standard armchair (approximately 46 cm seat height), walk a distance of 3 m as fast as they could safely, turn around, walk back to the chair, and sit down. The time taken to complete the task was recorded in seconds using a stopwatch. A single practice trial was permitted, followed by two recorded trials, with the average of the two trials used for the analysis. Established cut-off thresholds of >10.0 s, >12.0 s, and >13.5 s were applied to categorise the fall risk [20,21].

#### 2.3.2. Falls Efficacy Scale-International (FES-I)

The FES-I was used to quantify the participants’ self-reported fear of falling during 16 daily activities [19]. The participants rated their concern about falling for each activity on a 4-point Likert scale, ranging from 1 (not at all concerned) to 4 (very concerned). The cut-off scores used were FES-I concern categories: low concern (<20) vs. moderate/high concern (≥20), and low+moderate concern (≤27) vs. high concern (>27) [22]. The thresholds used to classify the participants according to their level of concern about falling were: low concern (<20), moderate concern (20–27), and high concern (>27).

### 2.4. Experimental Protocol

**Figure 2:**
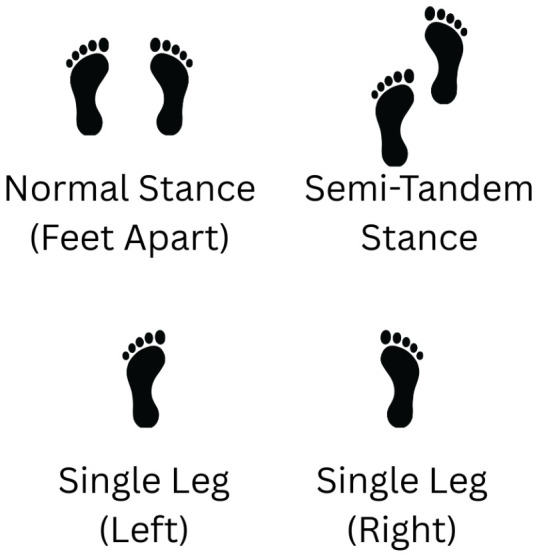
Schematic representation of the foot placements utilised during the balance testing protocol: normal stance (feet apart), semi-tandem stance, and single-leg stances on the left and right foot.

The participants performed static balance assessments across six stance conditions (Figure 2):

1. Normal stance, feet shoulder-width apart, eyes open;
2. Normal stance, feet shoulder-width apart, eyes closed;
3. Semi-tandem stance, right foot forward, eyes open;
4. Semi-tandem stance, right foot forward, eyes closed;
5. Single-leg stance (left foot), eyes open;
6. Single-leg stance (right foot), eyes open.

These six conditions were selected with a presumed range of postural difficulty by manipulating the base of support (normal, semi-tandem, or single-leg) and visual input (eyes open versus eyes closed), factors known to systematically influence the CoP sway in older adults [23,24]. Each stance was performed once for 20 s to avoid any learning effect and reduce fatigue [24]. A 3-s adjustment period at the start of each trial was excluded to allow the participants to stabilise. The participants were instructed to stand as still as possible with their arms relaxed at their sides while focusing on a fixed point during the eyes-open conditions. A 30-s rest period was provided between trials to minimise fatigue. An assessor remained nearby to ensure participant safety, particularly during the single-leg stance conditions. All the participants completed trials in the same testing environment under consistent lighting and flooring conditions [14]. The BM was placed on top of the FP for simultaneous recording using a 3-s countdown on the BM desktop application as a trigger for synchronisation.

### 2.5. Data Processing and Feature Extraction

Postural sway signals were extracted from both the BM and FP in accordance with previous literature [23,24]. The BM provides a one-dimensional unitless sway signal reflecting the plantar pressure distribution. Because the FP records the CoP in both the anterior-posterior (AP) and medial-lateral (ML) directions, we derived the resultant distance $CoP_{RD}$ from these two components to obtain a single scalar measure of the overall sway magnitude for comparison with the BM output. The $CoP_{RD}$ was calculated to quantify the overall sway magnitude, as defined by the following equation:

(1) $$CoP_{RD}=\sqrt{CoP_{AP_{i}}^{2}+CoP_{ML_{i}}^{2}}$$

The following sway features were extracted:

Sway Mean: The sway mean represents the average COP position over the trial and reflects the distribution of plantar pressure across the support surface. Deviations in the mean COP coordinates can indicate bilateral weight-distribution asymmetry and may serve as a clinical indicator of pathologies associated with postural imbalances [24].

(2) $$Mean\;Sway=\frac{1}{n}\sum_{i=1}^{n}X_{i}$$

Sway RMS: The sway RMS is the square root of the mean of the squared COP values and serves as a variability index of the COP displacement magnitude. When the mean COP is approximately zero, the RMS and the standard deviation yield equivalent estimates. The RMS has demonstrated reliable discrimination between younger and older adults and between healthy individuals and those with neurological pathologies [24].

(3) $$RMS\;Sway=\sqrt{\frac{1}{n}\sum_{i=1}^{n}X_{i}^{2}}$$

Sway Path: The sway path quantifies the total cumulative COP displacement over the trial duration. Shorter path lengths reflect greater postural stability, and this measure is considered a valid outcome across a range of populations and balance conditions [24].

(4) $$Sway\;Path=\sum_{i=1}^{n-1}\sqrt{{\left(X_{i+1}-X_{i}\right)}^{2}}$$

Sway Range: The sway range is the peak-to-peak amplitude of COP displacement along the measurement axis. Larger values correspond to poorer postural stability. This parameter has been used to characterise postural deficits in clinical populations, including individuals with neuromotor disorders [24].

(5) Sway Range=Max(X)−Min(X)

Sway Velocity: The sway velocity normalises the total COP path length by trial duration and reflects the net neuromuscular activity required to maintain an upright stance. Lower values indicate more efficient postural control. The COP velocity has been identified as one of the most sensitive and reliable parameters for distinguishing postural control across age groups and neurological conditions [24].

(6) $$Sway\;Velocity=\frac{Sway\;Path}{T}$$

Sway Variance: The sway variance quantifies the spread of COP positions around the trial mean, providing a measure of the postural oscillation magnitude. As the second central statistical moment of COP displacement, it is sensitive to differences in postural control between healthy individuals and clinical populations [24].

(7) $$\mathrm{Sway}\;\mathrm{Variance}=\frac{1}{n-1}\sum_{i=1}^{n}{\left(X_{i}-\bar{X}\right)}^{2}$$

Area95: The 95% ellipse area estimates the total region of COP excursion, encompassing 95% of the recorded data points, and serves as a global index of postural performance, with smaller values reflecting greater postural stability [24]. For the force plate, a bivariate prediction ellipse is fitted to the AP and ML COP time series using the *F*-distribution critical value. For the Balance Mat, which yields a univariate resultant displacement signal, an equivalent one-dimensional 95% confidence interval is used in place of the bivariate ellipse.

(8) $$\mathrm{Area}95_{\mathrm{FP}}=\pi \times F_{\left(0.05;2,n-2\right)}\times SD_{AP}\times SD_{ML}$$

(9) $$\mathrm{Area}95_{\mathrm{BM}}=2\times 1.96\times SD\left(X\right)$$

where $X_{i}$ represents the sway signal at sample *i* and *T* represents the trial duration.

These parameters are commonly used in postural stability analyses and fall-risk assessments [23,24]. The sway velocity and sway path are widely regarded as the most reliable and sensitive indicators of age-related changes in postural steadiness [23]. RMS sway provides a robust measure of the variability in the CoP displacement and is sensitive to ageing [16,25]. The sway range identifies the distance between the maximum and minimum. Higher values across these sway features indicate increased postural instability and a significantly elevated risk of falls [23,24].

### 2.6. Statistical Analysis

All statistical analyses were conducted using Python (v3.12.0, https://www.python.org/) and IBM SPSS Statistics v31.0.1.0 (IBM Corp., Armonk, NY, USA). Data normality was assessed using the Shapiro–Wilk test. As most sway variables were non-normally distributed, non-parametric statistical methods were employed. The association between the BM and FP was evaluated using Spearman’s rank correlation coefficient (ρ). The correlation strength was interpreted as very weak: <0.20, weak: 0.20–0.39, moderate: 0.40–0.59, strong: 0.60–0.79, or very strong: >0.80 [26].

**Figure A1:**
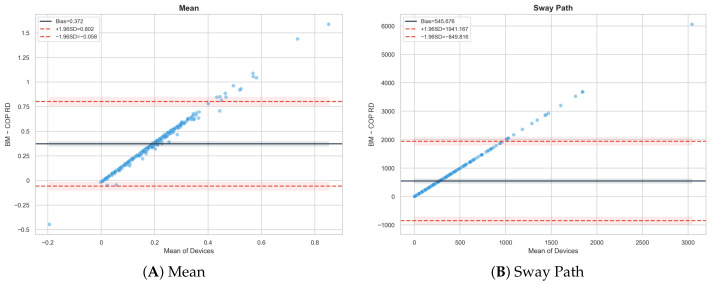
Representative Bland-Altman plots for uncalibrated BM versus FP (${\mathrm{CoP}}_{\mathrm{RD}}$).

The absolute agreement between devices was assessed using two-way random-effects intraclass correlation coefficients ($ICC_{2,1}$). The ICC values were interpreted as poor: <0.50, moderate: 0.50–0.75, good: 0.75–0.90 or excellent: >0.90 [27]. The agreement was further evaluated using the root mean square error (RMSE), standard error of measurement (SEM) and minimal detectable change (MDC). Bland–Altman plots were generated to visually assess the systematic and proportional bias (Figure A1). Where proportional bias was identified, simple linear regression models were applied to calibrate the BM outputs against FP measurements. Calibration equations were derived using a training dataset (70%) and evaluated on a held-out test dataset (30%) to minimise overfitting.

To evaluate whether the BM can discriminate between participants classified as a high vs. low fall risk, ROC curves were constructed. Risk classifications were based on cut-off scores from the TUG score, FES-I score and fall history. ROC curves graphically represent the trade-off between a test’s true positive rate (sensitivity) and false positive rate (1-specificity) across all possible decision thresholds. Specifically, the sensitivity reflects the device’s ability to correctly identify individuals with a true underlying balance deficit or fall risk, whereas the specificity reflects its ability to correctly designate healthy, non-risk individuals [28,29]. The overall discriminative capacity of each sway feature was evaluated using the AUC, which estimates the probability that the device will correctly rank a randomly selected high-risk subject higher than a randomly selected low-risk subject. Following standard clinical thresholds, the AUC values were interpreted as poor (0.60–0.69), fair (0.70–0.79), considerable (0.80–0.89) or excellent (≥0.90) [29]. To identify the optimal diagnostic cut-off for classifying participants into binary clinical risk categories, a sensitivity-prioritised approach was used. As the BM is intended for screening, the final cut-off was selected from thresholds with a sensitivity ≥ 0.75, choosing the one that maximised the specificity [29]. To further evaluate the diagnostic performance, confusion matrices were generated for each clinical classification. These matrices illustrate the absolute frequencies of true positive, true negative, false positive, and false negative classifications at the selected optimal cutpoints. These diagnostic metrics were computed to determine if the continuous BM sensor data could accurately classify participants according to the retrospective fall history, specific TUG completion time thresholds, and FES-I concern levels.

## 3. Results

### 3.1. Participant Characteristics

A total of 56 community-dwelling older adults participated in the study. The participant demographic characteristics, anthropometric data, and baseline clinical measures are presented in Table 1. Based on their retrospective fall history within the previous 12 months, participants were classified into fallers and non-fallers. Their TUG performance and FES-I scores were also recorded for clinical classification into high and low risk based on the TUG time and high, moderate and low concern based on the FES-I scores. Overall, the cohort had a mean age of 75.29±6.27 years and was predominantly female (76.8%). Participants classified retrospectively as fallers (n=12) generally exhibited higher mean TUG times (11.55±4.29 s) and FES-I scores (28.92±8.61) compared to non-fallers (n=44, TUG: 6.88±1.85 s, FES-I: 20.34±4.66).

### 3.2. Descriptive Analysis of Postural Sway

Postural sway features were extracted from the BM across the pooled stance conditions. Due to the high degree of inter-individual variability and the presence of positive skewness in the sway data (indicating a subset of highly unstable trials), both the mean and median values are reported to provide a comprehensive representation of the central tendency. The descriptive statistics for the extracted sway features are detailed in Table 2.

### 3.3. Technical Validity Between BM and FP

#### 3.3.1. Association Analysis

The Spearman correlation coefficients between BM sway features and the corresponding FP CoP measurements across all stance conditions are presented in Table 3. The sway path/velocity demonstrated the strongest association (r=0.85, p<0.001). However, the mean sway showed the weakest correlation (r=0.39, p<0.001). Since T was constant at 20 s across all trials, the sway velocity equalled the sway path/20, and both parameters yielded numerically identical correlation, ICC, and AUC values. All ICC, RMSE, SEM, and MDC values were derived from the held-out test set (30% of trials). The corresponding training-set metrics are provided in Table A2.

#### 3.3.2. Agreement Analysis

The absolute agreement between the calibrated BM and FP varied from poor to excellent depending on the extracted feature. The sway path/velocity demonstrated the highest absolute agreement, achieving excellent ICC values of 0.93 alongside the lowest relative measurement errors (Table 3). The mean sway demonstrated a poor agreement (ICC=0.30). To correct for systematic scaling differences as observed in Figure A1, linear regression calibration equations were derived specifically for the valid resultant distance ($CoP_{RD}$) sway features (Table 4). Spearman correlation coefficients and ICC values were also computed separately within each of the six stance conditions using only the single trial per patient recorded for that stance. The results are summarised in Table A1.

### 3.4. Clinical Discriminative Validity

**Figure 3:**
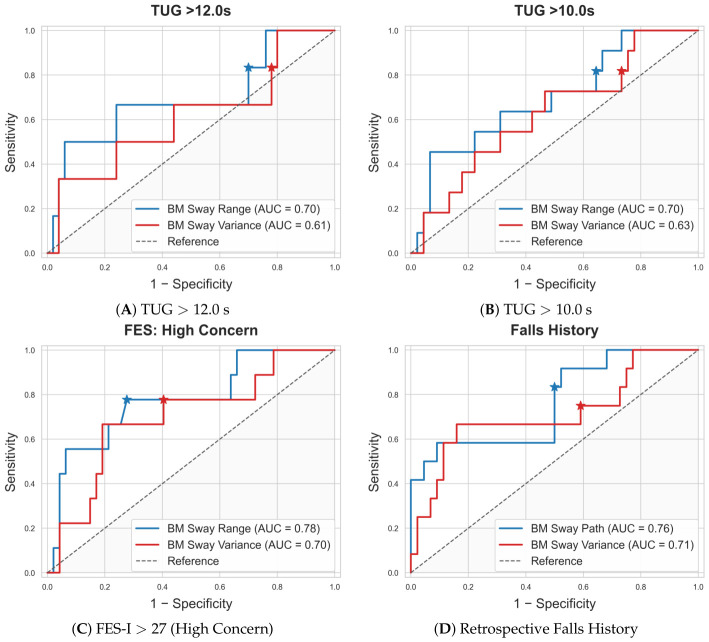
ROC curves demonstrating the diagnostic accuracy of the BM across four distinct clinical risk classifications.

Table 5 details the diagnostic accuracy of the BM and FP for identifying clinical fall risk thresholds using an ROC analysis. For both the FP and BM, all valid sway features were evaluated across all clinical thresholds. The AUC values for every parameter are reported in Table A4. The parameter with the highest AUC for each clinical threshold is reported in Table 5. For the BM, the overall diagnostic accuracy ranged from poor for predicting a low FES-I concern (AUC=0.67) to acceptable for predicting a high FES-I concern (AUC=0.78). Across all clinical benchmarks, the reference FP achieved a higher diagnostic ceiling, peaking at an excellent classification accuracy for the secondary TUG > 10.0 s threshold (AUC=0.90). Figure 3 visually plots the ROC curves and AUC values for the raw BM variables across four distinct clinical outcomes. The values in Table 5 represent the highest AUC selected post hoc across all sway features (Table A4) without correction for multiple comparisons, and should be interpreted as exploratory.

**Figure 4:**
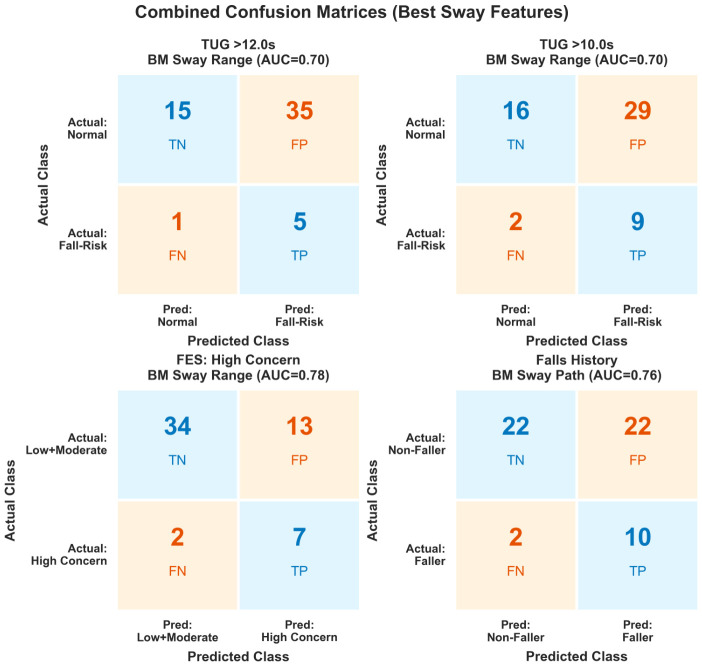
Confusion matrices demonstrating the classification performance of the best-performing Balance Mat sway features across four clinical risk thresholds.

**Figure 5:**
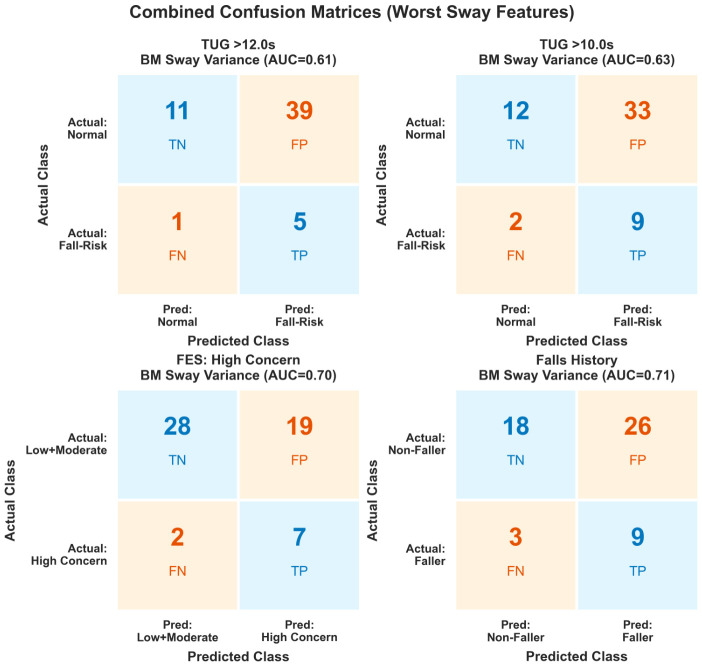
Confusion matrices demonstrating the classification performance of the worst-performing Balance Mat sway feature (sway variance) across four clinical risk thresholds.

Figure 4 and Figure 5 present the confusion matrices for the best- and worst-performing sway features, respectively. For the best features, such as the sway range and sway path, the Balance Mat correctly identified most high-risk individuals, though a notable number of false positives remained. The worst-performing feature, the sway variance, produced substantially more false positives across all clinical thresholds and reduced the overall classification accuracy.

To assess whether the pooled estimates were inflated by stance conditions, Spearman r and ICC(2,1) were computed separately within each of the six individual stance conditions, using a single trial per participant per condition. These within-condition results are presented in Table A1.

## 4. Discussion

This study assessed the BM for measuring postural sway in older adults by comparing it with the reference FP, TUG, FES-I, and the fall history. On comparison with the FP, the BM performed differently depending on the sway feature. It showed a weak to very strong correlation, from r = 0.39 for the mean sway to r = 0.85 for the sway path/velocity. After calibration, the agreement was excellent for the sway path/velocity (ICC = 0.93) and good for Area95 and RMS (ICC>0.75), but poor for the mean sway (ICC = 0.30), so the mean sway was excluded from further analysis. For fall-risk classification, the calibrated BM showed a fair accuracy for the retrospective fall history and a high FES-I concern (AUC 0.76–0.78), a fair accuracy for the TUG thresholds (AUC 0.70), and a poor accuracy for the FES-I low-concern threshold (AUC = 0.67). Overall, our findings partly support the hypothesis. The BM is not a direct FP substitute or a standalone diagnostic tool. Instead, it is best used as a screening tool. Sway features should be combined with the fall history and standard clinical tests to identify older adults needing a full fall assessment, and all interpretations should be made within this construct validity framework.

The BM performed better for the sway path/velocity, which reflect the total movement over time. The sway path/velocity, Area95, RMS, and variance correlated more strongly with the FP than the mean sway. This means that there is a more granular evaluation of the person’s balance as evidenced by a longer sway path/velocity associated with poorer balance. In contrast, the mean sway is less informative, as it loses that granular information and the individual variance, which provides a less accurate assessment of the individual’s balance. Most sway assessments instead rely on sway features such as the sway path/velocity, range, and area [14,30,31]. This is consistent with our earlier evaluation in younger adults [15], which showed a similar pattern and strength of correlations, together with a proportional bias that improved following calibration. Ghahramani et al. [14] reported similar strong associations between the BM and inertial sensors for the sway path/velocity, range and RMS in older adults, supporting the BM’s use as a practical tool for quantifying sway magnitude. However, it is important to note that the correlation reflects proportional tracking rather than strict measurement equivalence. Therefore, the BM is better viewed as a relative measure of sway than as a direct FP substitute. The stance-specific ICC ranged from 0.03 to 0.95 (Table A1), indicating that the pooled agreement in Table 3 does not reflect the performance uniformly across stances. This variability suggests that pooled agreement metrics should be interpreted with reference to the condition-specific data in Table A1.

Despite strong relative correlations, systematic scaling differences were observed between the BM and FP sway measures. The measurement differences increased linearly in magnitude with the mean of the two measures. To address this bias, linear calibration models were applied to align the BM outputs with the FP reference standard. Applying these linear models improved the measurement comparability, as reflected in the agreement analysis shown in Table 3. Similar calibration needs have been reported when portable balance tools are compared with the reference FP. Leach et al. [32] found that a linear correction was necessary to align Wii Balance Board measures with the FP. Sturnieks et al. [31] also observed systematic bias between a portable balance platform (Swaymeter) and the FP and recommended calibration equations to improve interchangeability. Our analysis demonstrated that the AUC, sensitivity, and specificity remained identical for both raw and calibrated BM data, with only the clinical cutoff points shifting (Table 5). This indicates that calibration is only strictly necessary when direct interchangeability with FP data is required. Once these population norms have been established, clinicians will be able to interpret raw BM scores directly, without the need for calibration equations [31].

The BM demonstrated a fair diagnostic accuracy for identifying the fall risk. These results must be interpreted within the context of the clinical benchmarks used to evaluate it. Across the primary clinical references, the AUC values ranged from 0.70 to 0.78 for the FES-I, TUG, and fall history, dropping to 0.67 for the FES-I low-concern category. An AUC of 0.70–0.78 means that the BM correctly ranks a random high-risk individual above a low-risk one in 70–78% of comparisons. Additionally, confusion matrices showed that even the best sway features produced a notable number of false positives. This fair classification performance is expected, as the reference clinical tests themselves have a limited predictive accuracy. When used alone, both the FES-I and TUG typically produce AUCs between 0.5 and 0.7 and show a highly variable sensitivity and specificity [33,34], and the retrospective fall history is subject to recall bias. Because individual clinical measures are often insufficient, guidelines suggest combining the fall history, self-report scales, and performance-based tests [35,36,37]. At a sensitivity-prioritised cut-off (0.83 sensitivity, 0.50 specificity against falls history), the BM identifies four in five fallers while flagging about half of non-fallers for further assessment. Therefore, the primary clinical value of the BM is not as a standalone diagnostic test, but as a rapid screening tool to flag older adults who require a more comprehensive fall assessment.

The diagnostic performance of the BM is similar to that of other portable balance devices. For example, Howcroft et al. [38] evaluated the Wii Balance Board in older adults and reported comparable classification accuracies (62–67%), with a 67–82% sensitivity and 57–60% specificity. Furthermore, Ghahramani et al. [39] demonstrated that the accuracy of wearable sensors depends heavily on the specific standing task, finding AUCs that ranged from 0.62 during eyes-closed standing to 0.90 during tandem standing. Prospective studies have also reported a fair predictive accuracy (AUC around 0.70) when using isolated postural sway measures [40]. A systematic review and meta-analysis found that several CoP variables can help distinguish fallers from non-fallers [41]. Another study by Edginton et al. [42] showed that the performance is strongest when multiple variables are considered together rather than when a single measure is used. In this context, the BM offers an immediate and objective measure of the postural sway that performs comparably to other portable systems. While portable tools provide valuable objective data, isolated single measures rarely offer a complete clinical picture. Therefore, BM assessments should be integrated with the fall history and other clinical tests.

The older adult sample also showed wide variability in sway, as observed in the descriptive statistics. Some participants showed a much larger sway than others, which is common in ageing cohorts and is exactly why continuous sway features are useful. Mancini et al. [43] demonstrated that postural sway increases with age, but also varies widely according to the functional ability and fall history. Delmas et al. [44] similarly reported broad sway distributions in healthy ageing, with some individuals showing disproportionately high values. Given this wide variability, clinical interpretation should prioritise continuous sway features, as binary categorisation risks the loss of valuable individual data.

### Limitations and Future Directions

Although the BM performed reasonably well, the following limitations should be taken into consideration. First, the study used the retrospective fall history and current clinical tests as the reference standards, and both have known limits for predicting future falls. Second, the ROC analyses were not part of the original sample-size calculation, and several subgroups contained few positive cases; therefore, these estimates should be interpreted as an exploratory model. Third, the device was tested only under quiet-standing conditions, so its performance during dynamic tasks or more demanding sensory or dual-task conditions remains unknown. Fourth, the participants were community-dwelling older adults from a convenience sample, and the experiments were conducted at a single centre under controlled laboratory conditions, which may not reflect real-world settings. Additionally, the BM measures the static standing balance, whereas the TUG assesses dynamic functional mobility. The comparisons, therefore, reflect construct validity rather than clinical interchangeability.

Future studies should also examine the Balance Mat performance during dynamic tasks, use prospective fall follow-up, include larger and more balanced samples, and incorporate more challenging standing tasks. They should also test whether combining BM sway variables with age, fall history, mobility, and cognitive measures improves the prediction. This would show whether the BM is best used as a screening device, a monitoring tool, or part of a broader fall-risk model.

## 5. Conclusions

The BM showed useful potential, but it is not yet a full replacement for the FP. It measured sway well for most parameters, especially the sway path/velocity and Area95, and calibration improved its agreement with the FP values. However, the mean sway remained weak, and the device showed a fair accuracy for fall-risk classification, so it should not be used as a standalone clinical test. The within-stance agreement for the sway path/velocity ranged from ICC = 0.44 to 0.88 (Table A1), and the pooled value should not be interpreted as the uniform performance across all stance conditions. Overall, the BM is best used as a portable tool for sway and should be used in combination with the fall history and clinical tests.

## Figures and Tables

**Table 1:** Demographic and clinical characteristics of the older adult cohort, grouped by retrospective fall history.

Variable	Category/Metric	Overall (N=56)	Non-Fallers (n=44)	Fallers (n=12)
Continuous Variables (Mean ± SD [Range])				
Age (Years)		75.29±6.27 [65.0–88.0]	74.70±6.06 [65.0–88.0]	77.42±6.82 [65.0–88.0]
Height (cm)		163.99±10.23 [142.0–183.0]	162.68±9.67 [142.0–183.0]	168.76±11.22 [149.9–183.0]
Weight (kg)		69.94±14.77 [44.0–104.0]	68.32±14.32 [44.0–104.0]	75.92±15.49 [54.0–100.0]
TUG Time (s)		7.88±3.18 [4.2–21.7]	6.88±1.85 [4.2–14.3]	11.55±4.29 [5.7–21.7]
FES-I Score		22.18±6.66 [16.0–42.0]	20.34±4.66 [16.0–39.0]	28.92±8.61 [19.0–42.0]
Categorical Variables (*n* [%])				
Gender	Female	43 (76.8%)	35 (79.5%)	8 (66.7%)
	Male	13 (23.2%)	9 (20.5%)	4 (33.3%)
Falls History	0 Falls	44 (78.6%)	44 (100.0%)	-
	1 Fall	8 (14.3%)	-	8 (66.7%)
	2 Falls	4 (7.1%)	-	4 (33.3%)

**Table 2:** Descriptive statistics of postural sway features for the older adult cohort, comparing the Balance Mat and force plate across pooled stance conditions.

Sway Feature	Cohort	Mean ± SD	Median	Range
Balance Mat				
Sway Mean	Overall (n=56)	0.38±0.07	0.38	0.24–0.53
	Non-Fallers (n=44)	0.37±0.06	0.36	0.24–0.53
	Fallers (n=12)	0.42±0.07	0.42	0.32–0.51
RMS	Overall	5.47±2.82	4.83	1.38–16.20
	Non-Fallers	4.78±2.03	4.75	1.38–11.70
	Fallers	8.00±3.85	8.08	3.36–16.20
Sway Path	Overall	546.37±264.12	506.42	122.83–1603.60
	Non-Fallers	479.96±180.67	484.67	122.83–846.83
	Fallers	789.88±372.67	749.00	370.60–1603.60
Sway Range	Overall	36.36±19.42	32.17	11.50–100.20
	Non-Fallers	31.62±14.43	30.67	11.50–88.33
	Fallers	53.75±25.52	57.83	21.40–100.20
Sway Velocity	Overall	27.32±13.21	25.32	6.14–80.18
	Non-Fallers	24.00±9.03	24.23	6.14–42.34
	Fallers	39.49±18.63	37.45	18.53–80.18
Sway Variance	Overall	82.19±115.57	47.81	3.71–758.11
	Non-Fallers	59.22±62.66	45.01	3.71–341.99
	Fallers	166.40±203.93	128.40	17.50–758.11
Area95	Overall	33.53±17.39	29.59	8.30–99.65
	Non-Fallers	29.28±12.50	29.09	8.30–71.92
	Fallers	49.11±23.75	49.63	20.47–99.65
Force Plate (${\mathrm{COP}}_{\mathrm{RD}}$)				
Sway Mean	Overall	0.013±0.004	0.012	0.007–0.024
	Non-Fallers	0.012±0.004	0.011	0.007–0.023
	Fallers	0.015±0.005	0.013	0.008–0.024
RMS	Overall	0.015±0.005	0.013	0.007–0.029
	Non-Fallers	0.014±0.004	0.013	0.007–0.027
	Fallers	0.019±0.007	0.016	0.009–0.029
Sway Path	Overall	0.816±0.396	0.746	0.443–3.439
	Non-Fallers	0.729±0.134	0.728	0.443–1.064
	Fallers	1.134±0.757	0.935	0.671–3.439
Sway Range	Overall	0.043±0.037	0.036	0.014–0.283
	Non-Fallers	0.036±0.015	0.034	0.014–0.083
	Fallers	0.067±0.071	0.045	0.022–0.283
Sway Velocity	Overall	0.041±0.020	0.037	0.022–0.172
	Non-Fallers	0.036±0.007	0.036	0.022–0.053
	Fallers	0.057±0.038	0.047	0.034–0.172
Sway Variance	Overall	0.000±0.000	0.000	0.000–0.002
	Non-Fallers	0.000±0.000	0.000	0.000–0.000
	Fallers	0.000±0.000	0.000	0.000–0.002
Area95	Overall	0.044±0.022	0.037	0.015–0.138
	Non-Fallers	0.039±0.016	0.036	0.015–0.087
	Fallers	0.061±0.033	0.053	0.026–0.138

**Table 3:** Association (Spearman correlation) and absolute agreement (ICC, RMSE, SEM, MDC) between the calibrated BM and FP for the resultant distance ($CoP_{RD}$) sway features. Absolute agreement (ICC) and error metrics (RMSE, SEM and MDC) were derived from the test dataset (30% of trials).

Sway Feature	Spearman *r*	ICC (2,1)	RMSE	SEM	MDC	Agreement Grade
Mean	0.39 **	0.30	0.0085	0.0078	0.0217	Poor
RMS	0.79 **	0.80	0.0073	0.0052	0.0145	Good
Sway Path	0.85 **	0.93	0.2485	0.1412	0.3912	Excellent
Sway Range	0.68 **	0.72	0.0407	0.0224	0.0622	Moderate
Sway Velocity	0.85 **	0.93	0.0124	0.0071	0.0196	Excellent
Area95	0.81 **	0.80	0.0286	0.0194	0.0537	Good
Sway Variance	0.83 **	0.70	0.0003	0.0001	0.0004	Moderate

** Correlation was significant at the 0.001 level. ICC agreement grade interpretation: <0.50, poor; 0.50–0.75, moderate; 0.75–0.90, good; >0.90, excellent.

**Table 4:** Calibration parameters and Bland-Altman numerical summary for Balance Mat measurements ($CoP_{RD}$).

Sway Feature	Calibration		Bland-Altman Agreement		
	Slope	Intercept	Mean Bias	Lower 95% LoA	Upper 95% LoA
RMS	0.0016	0.0064	0.00091	−0.01337	0.01519
Sway Path	0.0011	0.1902	0.00226	−0.48716	0.49168
Sway Range	0.0014	−0.0064	0.00428	−0.07543	0.08399
Sway Velocity	0.0011	0.0095	0.00011	−0.02436	0.02458
Area95	0.0011	0.0084	0.00271	−0.05328	0.05870
Sway Variance	0.0000	0.0000	0.00004	−0.00045	0.00053

Note: Calibration equation format: $BM_{calib}=\left(\mathrm{Slope}\times BM_{raw}\right)+\mathrm{Intercept}$.

**Table 5:** Diagnostic accuracy, 95% confidence intervals, and likelihood ratios of the best-performing variables for identifying clinical fall risk thresholds. Balance Mat sway features are compared to the validated resultant distance ($CoP_{RD}$).

Clinical Reference (Sample Sizes)	Best Sway Feature	AUC [95% CI]	Cutpoint ^a^	Sens	Spec	LR+	LR−
Balance Mat (BM)-Resultant Distance Only							
FES-I: Low Concern (n=30 Mod/High, n=26 Low)	BM RD Sway Velocity	0.67 [0.53–0.82]	22.17/0.04	0.77	0.58	1.81	0.40
FES-I: High Concern (n=9 High, n=47 Low + Mod)	BM RD Sway Range	0.78 [0.60–0.96]	39.00/0.05	0.78	0.72	2.81	0.31
TUG >12.0 s (n=6 High Fall Risk, n=50 Low Fall Risk)	BM RD Sway Range	0.70 [0.42–0.98]	62.83/0.03	0.83	0.30	1.19	0.56
TUG >10.0 s (n=11 High Fall Risk, n=45 Low Fall Risk)	BM RD Sway Range	0.70 [0.52–0.88]	60.83/0.03	0.82	0.36	1.27	0.51
Fall History (n=12 Fallers, n=44 Non-Fallers)	BM RD Sway Path	0.76 [0.60–0.93]	708.33/0.72	0.80	0.51	1.64	0.39
Force Plate-Reference Standard							
FES-I: Low Concern (n=30 Mod/High, n=26 Low)	$FP_{AP}$ RMS	0.78 [0.66–0.91]	0.01	0.80	0.77	3.47	0.26
FES-I: High Concern (n=9 High, n=47 Low + Mod)	$FP_{AP}$ Area95	0.79 [0.62–0.95]	0.05	0.78	0.64	2.15	0.35
TUG >12.0 s (n=6 High Fall Risk, n=50 Low Fall Risk)	$FP_{ML}$ RMS	0.93 [0.85–1.00]	0.02	0.83	0.86	5.95	0.19
TUG >10.0 s (n=11 High Fall Risk, n=45 Low Fall Risk)	$FP_{ML}$ Sway Range	0.90 [0.81–0.98]	0.05	0.91	0.87	6.82	0.11
Fall History (n=12 Fallers, n=44 Non-Fallers)	$FP_{AP}$ Sway Path	0.84 [0.70–0.98]	0.83	0.80	0.76	3.28	0.27

AUC interpretation: ≥0.90, excellent; 0.80–0.89, considerable; 0.70–0.79, fair; 0.60–0.69, poor. Mean sway was excluded from the main analysis owing to a poor calibration agreement with the force plate reference (ICC(2,1) ^a^). BM cutpoints are displayed as raw/calibrated values. The diagnostic metrics (AUC, Sens, Spec, LR+, LR−) were identical for both raw and calibrated values. To minimise missed cases in high-risk screening, optimal cutpoints were selected using a sensitivity-prioritised strategy (target sensitivity, ≥0.75). The TUG > 13.5 s threshold was excluded due to an extreme class imbalance.

## Data Availability

All the extracted datasets, coding frameworks, and statistical analysis scripts are publicly available at https://github.com/ShresthaAvi, (accessed on 15 June 2026).

## Abbreviations

The following abbreviations are used in this manuscript:

BM	Balance Mat
FP	Force Plate
CoP	Centre of Pressure
AP	Anterior-Posterior
ML	Medial-Lateral
RD	Resultant Distance
FES-I	Falls Efficacy Scale-International
TUG	Timed Up and Go
ROC	Receiver Operating Characteristic
AUC	Area Under the Curve

## Appendix A

**Table A1:** Stance-wise technical validity metrics.

Stance Condition	Metric	Spearman *r*	*p*-Value	ICC(2,1)
Normal EO	Sway Path/Velocity	0.999	<0.001	0.44
	Area95	0.48	<0.001	0.22
	Sway Range	0.51	<0.001	0.08
	RMS	0.26	0.057	0.12
	Sway Variance	0.48	<0.001	0.03
	Mean	0.22	>0.05	0.10
Normal EC	Sway Path/Velocity	1.00	<0.001	0.57
	Area95	0.33	0.016	0.24
	Sway Range	0.31	0.023	0.12
	RMS	0.34	0.014	0.24
	Sway Variance	0.34	0.013	0.12
	Mean	0.24	>0.05	0.16
Semi-Tandem EO	Sway Path/Velocity	0.998	<0.001	0.78
	Area95	0.62	<0.001	0.55
	Sway Range	0.51	<0.001	0.34
	RMS	0.39	0.004	0.36
	Sway Variance	0.64	<0.001	0.38
	Mean	0.14	>0.05	0.08
Semi-Tandem EC	Sway Path/Velocity	0.999	<0.001	0.88
	Area95	0.61	<0.001	0.46
	Sway Range	0.58	<0.001	0.36
	RMS	0.58	<0.001	0.39
	Sway Variance	0.60	<0.001	0.32
	Mean	0.27	>0.05	0.08
Single-Leg L EO	Sway Path/Velocity	0.999	<0.001	0.76
	Area95	0.37	0.007	0.82
	Sway Range	0.33	0.016	0.58
	RMS	0.42	0.002	0.82
	Sway Variance	0.37	0.007	0.95
	Mean	0.17	>0.05	0.06
Single-Leg R EO	Sway Path/Velocity	1.00	<0.001	0.83
	Area95	0.80	<0.001	0.63
	Sway Range	0.75	<0.001	0.58
	RMS	0.79	<0.001	0.61
	Sway Variance	0.80	<0.001	0.45
	Mean	0.07	>0.05	0.08

Spearman *r* values ≥ 0.998 are reported to three decimal places to preserve the distinction between near-perfect and perfect agreement. ICC, intraclass correlation coefficient; EO, eyes open; EC, eyes closed; L, left; R, right.

**Table A2:** ICC(2,1), root mean square error (RMSE), standard error of measurement (SEM), and minimal detectable change (MDC) of calibrated Balance Mat sway metrics, reported for the 70% training set and 30% test set (${\mathrm{CoP}}_{\mathrm{RD}}$, resultant displacement axis).

Metric	Partition	ICC(2,1)	RMSE	SEM	MDC
Mean	Training	0.24	0.0105	0.0099	0.0273
	Test	0.30	0.0085	0.0078	0.0217
Sway Path	Training	0.83	0.5245	0.4014	1.1126
	Test	0.93	0.2485	0.1412	0.3912
Sway Velocity	Training	0.83	0.0262	0.0201	0.0556
	Test	0.93	0.0124	0.0071	0.0196
Area95	Training	0.80	0.0338	0.0262	0.0727
	Test	0.80	0.0286	0.0194	0.0537
RMS	Training	0.77	0.0088	0.0069	0.0192
	Test	0.80	0.0073	0.0052	0.0145
Sway Range	Training	0.65	0.0661	0.0543	0.1505
	Test	0.72	0.0407	0.0224	0.0622
Sway Variance	Training	0.85	0.0003	0.0003	0.0007
	Test	0.70	0.0003	0.0001	0.0004

ICC, intraclass correlation coefficient, calibrated vs. force plate reference; RMSE, root mean square error; SEM $=\mathrm{RMSE}\times \sqrt{1-\mathrm{ICC}}$; MDC $=1.96\times \sqrt{2}\times \mathrm{SEM}$ (95% level). Training and test ICC values differed by ≤0.10 for five of seven metrics; the larger discrepancy for the sway variance (0.85 vs. 0.70) is consistent with greater variability in the smaller test partition. The mean sway showed a poor calibration agreement (ICC(2,1) ≤ 0.30) and was therefore excluded from the diagnostic accuracy analysis (Table 5).

**Table A3:** Bootstrap stability of AUC estimates for the best-performing sway variable per clinical outcome and device (n=56; 1000 stratified resamples). Full AUC results for all variables are provided in Table A1.

Outcome	Device	Variable	AUC	DeLong 95 % CI	Boot 95 % CI	Boot SD	Sig
Fall History (Non-Faller n=44; Faller n=12)							
	BM Calibrated	Sway Path	0.76	[0.60–0.93]	[0.59–0.91]	0.08	**
	Force Plate (RD) *^a^*	Sway Path	0.78	[0.62–0.93]	[0.61–0.91]	0.08	***
FES-I: Low Concern (>19; Low n=26; Moderate + High n=30)							
	BM Calibrated	Sway Velocity	0.67	[0.53–0.82]	[0.52–0.81]	0.07	*
	Force Plate (RD) *^a^*	Sway Path	0.67	[0.52–0.81]	[0.52–0.81]	0.07	*
FES-I: High Concern (>27; Low + Moderate n=47; High n=9) ^†^							
	BM Calibrated	Sway Range	0.78	[0.60–0.96]	[0.59–0.94]	0.09	**
	Force Plate (RD) *^a^*	Sway Path	0.75	[0.60–0.90]	[0.59–0.88]	0.07	**
TUG > 12.0 s (n=6 Fall Risk; n=50 Normal) ^†^							
	BM Calibrated	Sway Range	0.70	[0.42–0.98]	[0.51–0.95]	0.12	ns
	Force Plate (RD) *^a^*	Sway Path	0.68	[0.45–0.91]	[0.51–0.89]	0.10	ns
TUG > 10.0 s (n=11 Fall Risk; n=45 Normal)							
	BM Calibrated	Sway Range	0.70	[0.52–0.88]	[0.53–0.85]	0.08	*
	Force Plate (RD) *^a^*	Sway Path	0.70	[0.54–0.86]	[0.54–0.84]	0.08	*

^†^ Positive class *n* < 10; estimates are exploratory. *^a^* Force plate bootstrap reflects the resultant displacement axis (${\mathrm{CoP}}_{\mathrm{RD}}$); the directional-axis results (${\mathrm{FP}}_{\mathrm{AP}}$, ${\mathrm{FP}}_{\mathrm{ML}}$) reported in Table 5 were not subject to bootstrap resampling. Boot 95% CI, 2.5th–97.5th percentile of bootstrap AUC distribution; Boot SD, standard deviation across resamples. Sig, DeLong *z*-test against $H_{0}$: AUC =0.50; *** *p* < 0.001, ** *p* < 0.01, * *p* < 0.05; ns, not significant. BM, Balance Mat; FES-I, Falls Efficacy Scale-International; TUG, timed up and go; RD, resultant displacement.

**Table A4:** AUC (DeLong *z*-test significance) for all sway variables by clinical outcome and device (n=56). Variables are ordered by BM-calibrated AUC within each outcome.

Variable	Fall History		FES-I Low		FES-I High ^†^		TUG > 12 s ^†^		TUG > 10 s	
	BM Cal	FP RD	BM Cal	FP RD	BM Cal	FP RD	BM Cal	FP RD	BM Cal	FP RD
Sway Path	0.76 **	0.78 ***	0.67 *	0.67 *	0.73 **	0.75 **	0.64	0.68	0.68 *	0.70 *
Sway Velocity	0.76 **	0.78 ***	0.67 *	0.67 *	0.73 **	0.75 **	0.64	0.68	0.68 *	0.70 *
Sway Range	0.76 **	0.71 *	0.66 *	0.64	0.78 **	0.68 *	0.70	0.62	0.70 *	0.67 *
Area95	0.75 **	0.72 *	0.66 *	0.64	0.76 **	0.67 *	0.66	0.62	0.68 *	0.69 *
RMS	0.75 **	0.72 *	0.66 *	0.63	0.76 **	0.66	0.66	0.63	0.68 *	0.69 *
Sway Variance	0.71 *	0.69 *	0.66 *	0.63	0.70 *	0.62	0.61	0.57	0.63	0.64
Mean *^a^*	0.70 *	0.69 *	0.64	0.61	0.80 ***	0.65	0.75 *	0.63	0.72 **	0.68 *

^†^ Positive class *n* < 10; estimates are exploratory. *^a^* Mean sway was excluded from the main analysis owing to a poor calibration agreement with the force plate reference (ICC(2,1) ≤ 0.30); values are included here for reference only. Sig, DeLong *z*-test against $H_{0}$: AUC = 0.50; *** *p* < 0.001, ** *p* < 0.01, * *p* < 0.05; unmarked, not significant. BM Cal, Balance Mat-calibrated (${\mathrm{CoP}}_{\mathrm{RD}}$); FP RD, force plate resultant displacement; FES-I Low, FES-I > 19 (low vs. moderate + high); FES-I High, FES-I > 27 (low + moderate vs. high).
